# Dimethylarginines in patients with intracerebral hemorrhage: association with outcome, hematoma enlargement, and edema

**DOI:** 10.1186/s12974-017-1016-1

**Published:** 2017-12-13

**Authors:** Hans Worthmann, Na Li, Jens Martens-Lobenhoffer, Meike Dirks, Ramona Schuppner, Ralf Lichtinghagen, Jan T. Kielstein, Peter Raab, Heinrich Lanfermann, Stefanie M. Bode-Böger, Karin Weissenborn

**Affiliations:** 1 Department of Neurology, Hannover Medical School, 30623 Hannover, Germany; 2 Department of Neurology, Beijing Tiantan Hospital, Capital Medical University, Beijing, China; 3 Department of Clinical Pharmacology, Otto-von-Guericke-University of Magdeburg, University Hospital, Magdeburg, Germany; 4 Department of Clinical Chemistry, Hannover Medical School, Hannover, Germany; 5 Department of Nephrology and Hypertension, Hannover Medical School, Hannover, Germany; 6 Medical Clinic V, Academic Teaching Hospital Braunschweig, Braunschweig, Germany; 7 Institute of Diagnostic and Interventional Neuroradiology, Hannover Medical School, Hannover, Germany

**Keywords:** ADMA, SDMA, Intracerebral hemorrhage, Outcome, Hematoma enlargement, Edema

## Abstract

**Background:**

Asymmetric dimethylarginine (ADMA)––the most potent endogenous NO-synthase inhibitor, has been regarded as mediator of endothelial dysfunction and oxidative stress. Considering experimental data, levels of ADMA and its structural isomer symmetric dimethylarginine (SDMA) might be elevated after intracerebral hemorrhage (ICH) and associated with clinical outcome and secondary brain injury.

**Methods:**

Blood samples from 20 patients with acute ICH were taken at ≤ 24 h and 3 and 7 days after the event. Nine patients had favorable (modified Rankin Scale (mRS) at 90 days 0–2) outcome, and 11 patients unfavorable outcome (mRS 3–6). Patients’ serum ADMA, SDMA, and L-arginine levels were determined by high-performance liquid chromatography–tandem mass spectrometry. Levels were compared to those of 30 control subjects without ICH. For further analysis, patients were grouped according to outcome, hematoma and perihematomal edema volumes, occurrence of hematoma enlargement, and cytotoxic edema as measured by computed tomography and serial magnetic resonance imaging.

**Results:**

Levels of ADMA––but not SDMA and L-arginine––were elevated in ICH patients compared to controls (binary logistic regression analysis: ADMA ≤ 24 h, *p* = 0.003; 3 days *p* = 0.005; 7 days *p* = 0.004). If patients were grouped according to outcome, dimethylarginines were increased in patients with unfavorable outcome. The binary logistic regression analysis confirmed an association of SDMA levels ≤ 24 h (*p* = 0.048) and at 3 days (*p* = 0.028) with unfavorable outcome. ADMA ≤ 24 h was increased in patients with hematoma enlargement (*p* = 0.003), while SDMA ≤ 24 h was increased in patients with large hematoma (*p* = 0.029) and perihematomal edema volume (*p* = 0.023).

**Conclusions:**

Our data demonstrate an association between dimethylarginines and outcome of ICH. However, further studies are needed to confirm this relationship and elucidate the mechanisms behind.

## Background

Intracerebal hemorrhage (ICH) is a detrimental disease with high morbidity and mortality [1]. Deterioration by secondary hematoma enlargement and perihematomal edema occurs frequently during the first days leading to a high rate of poor outcome underlining the need for effective therapy. Knowledge about molecular mechanisms being involved in these important complications that could facilitate therapeutic approaches is still sparse.

After ICH, thrombin and hemin, a hemoglobin metabolite, enter the brain parenchyma. Experimental data from cell culture models of microglia and cortical astrocytes indicate that these components lead to overexpression of inducible nitric oxide synthase (iNOS) and increased production of superoxide [2, 3]. Importantly, in ICH patients, levels of NO or NO metabolites were correlated with outcome. Chiang et al. showed an association of increased NO levels in cerebrospinal fluid with poor outcome at 6 months [4]. In contrast to this, Rashid et al. detected lower serum levels of NO metabolites in patients with poor outcome at discharge [5]. NO levels are differently influenced by dimetyhlarginines asymmetric dimetyhlarginine (ADMA) and its structural isomer symmetric dimethylarginine (SDMA).

Mono and dimethylarginines L-NG-monomethylarginine (N-NMMA), ADMA, and SDMA are generated by proteolysis from proteins with methylated arginine residues as catalyzed by protein arginine methyltransferases (PRMTs) [6, 7]. The structure of ADMA resembles L-arginine, which acts as the substrate of NOS to synthesize NO. For this reason, ADMA represents the most potent endogenous NOS inhibitor as it competes with L-arginine for NOS binding [8]. In acute ischemic stroke, it has been regarded as mediator of oxidative stress by inhibition and uncoupling of NO-synthases (for a review see [9]). Its structural isomer symmetric dimethylarginine (SDMA) inhibits production of NO levels differently through impairment of intracellular L-arginine uptake since L-arginine acts as the substrate of NOS [10]. SDMA increases the production of reactive oxygen species (ROS) as shown in monocytes [11].

By these mechanisms, dimethylarginines and L-arginine may contribute to the NO metabolism and oxidative stress also after ICH and might be involved in clinical outcome and secondary complications such as hematoma enlargement or perihematomal edema.

So far, dimethylarginine levels have been measured in only one study of ICH patients (*n* = 22) and did not differ from levels in control subjects [12]. However, the study by Wanby and colleagues leaves several questions unanswered since neither the time point of blood sampling has been specified nor have any clinical or radiological outcomes been analyzed in the study. Dimethylarginine levels may vary during the first days after ICH––similar to patients with ischemic stroke [13]. We expected a delayed increase in ADMA levels in ICH patients comparable to the findings in subarachnoid hemorrhage (SAH) patients [14, 15].

We aimed to investigate the temporal pattern of ADMA, SDMA, and L-arginine after ICH in regard to clinical outcome. In addition, we intended to examine the association of dimethylarginines with hematoma and perihematomal volumes and occurrence of hematoma enlargement and cytotoxic edema.

## Methods

### Study population

Twenty patients with primary ICH who presented within 24 h of symptom onset and were treated either at the stroke unit or the intensive care unit in the Department of Neurology at Hannover Medical School were enrolled. The patient cohort derived from a cohort of a former study [16]. Intracerebral hemorrhage was diagnosed by computed tomography (CT) scan or magnetic resonance tomography (MRI). Exclusion criteria were surgical ICH procedures, contraindication to MRI or refusal of participation. Thirty healthy subjects adjusted for sex and age served as controls. Patients and controls were assessed for age, sex, arterial hypertension, diabetes mellitus, estimated glomerular filtration rate (evaluated by CKD-EPI equation), hyperlipidemia, status of smoking, and treatment with anticoagulants and antiplatelets. In addition, in patients, baseline stroke severity (according to National Institutes of Health Stroke Scale Score (NIHSS) on admission) and clinical outcome (modified Rankin Scale (mRS) at 90 days) were obtained (favorable outcome mRS 0–2, unfavorable outcome mRS 3–6). For the definition of outcome groups, moderately and severely disabled patients as used in other studies [17] were combined in the unfavorable outcome group since in our patient cohort, the most severely disabled patients could not be included due to surgical procedures and impossibility of performance of serial MRI investigation.

The study was approved by the ethics committee of Hannover Medical School. Patients or a relative gave written informed consent.

### Blood collection and measurement of ADMA, SDMA, and L-arginine

Venous blood samples were taken at ≤ 24 h (median 12 h) and 3 and 7 days after the event. Serum was stored at − 80 °C until assayed. Serum ADMA, SDMA, and L-arginine were assessed blindly without knowledge of any of the clinical information using high-performance liquid chromatography–tandem mass spectrometry (HPLC–MS–MS) [18]. The lower limits of quantification for ADMA were 0.15 μmol/l, for SDMA were 0.20 μmol/l, and for L-arginine were 7.5 μmol/l. The inter-assay precision was 3.77%, and the intra-assay precision was 2.12% for ADMA, 3.86, and 2.83% for SDMA and 4.01 and 0.82% for L-arginine.

### Imaging protocol and analysis

On admission, CT was performed for clinical diagnosis. Magnetic resonance imaging (MRI) was conducted at ≤ 24 h (*n* = 18), 3 days (*n* = 18), and 7 days (*n* = 16) after the event, as feasible. For detection of hematoma and perihematomal edema volume by manual segmentation on 3D-fluid-attenuated-inversion recovery-data, ITK-SNAP analysis software was used. Cytotoxic edema was defined by area of elevated diffusion-weighted imaging (DWI)-b1000-signal and decreased apparent diffusion coefficient (ADC) value (by > 10% compared with mirror region of interest (ROI)) outside of hematoma on T2*- and DWIb0-sequences. The manually outlined area was confirmed by 3D-multiplanar localization using the image analysis software. Patients were grouped according to median hematoma volume considering the initial MRI. In two patients, no MRI was conducted at ≤ 24 h due to a severe clinical deficit on admission. In these patients, MRI at 3 days was used for categorization of hematoma volume. Hematoma enlargement was defined by increase of hematoma volume > 33% or 6.0 ml calculated from first imaging (CT or MRI) to follow-up MRIs. When hematoma volumes from CT and MRI were compared, MRI volumes were adapted as proposed by Burgess et al. (CT volume = 0.8* MRI volume) [19]. Patients without MRI at 3 or 7 days (*n* = 2) were excluded from this analysis. For analysis of patient groups according to perihematomal edema volume, the maximum values from MRI (≤ 24 h and 3 or 7 days) were used. In addition, patients were grouped for occurrence of cytotoxic edema as detected by MRI (≤ 24 h and 3 or 7 days).

### Statistical analysis

Statistical analysis was performed with the IBM SPSS Statistics 23. The data are presented as numbers and portion for categorical variables and median with interquartile range for continuous variables. The data were tested for statistically significant differences between patients and controls by Mann-Whitney *U* test for continuous data and Pearson chi-square for categorical data. The binary logistic regression analysis included ADMA levels and adjusted for co-variables age and estimated glomerular filtration rate (eGFR) using the method of backward stepwise. Within group comparisons of ADMA, SDMA and L-arginine levels at different time points were analyzed by Wilcoxon test. For the outcome analysis, patients were grouped into those with favorable and unfavorable outcome. In addition, Spearman rank correlation was performed between molecular marker levels and mRS at 90 days. To analyze the association of ADMA, SDMA, and L-arginine with imaging outcomes, patients were grouped according to hematoma and perihematomal edema volume and occurrence of hematoma enlargement and cytotoxic edema. For the analysis of clinical and imaging outcomes, the data were tested for statistically significant differences between patient groups by Mann-Whitney *U* test for continuous data and Pearson chi-square for categorical data. The binary logistic regression analysis tested ADMA and SDMA levels for association with clinical outcome including age and eGFR as co-variables (method of backward stepwise). A *p* value < 0.05 was considered to indicate statistical significance.

## Results

The study population consisted of 20 patients with ICH with a median age of 77 years (interquartile range 72–84). Clinical and demographical characteristics in patients and controls are shown in Table 1. Patients and controls did not significantly differ in regard to these parameters.

**Table 1:** Clinical characteristics of patients and controls

	ICH (*n* = 20)	Controls (*n* = 30)	*P*
Female	11 (55.0)	17 (56.7)	0.907
Male	9 (45.0)	13 (43.3)	
Age (years)	77 (72; 84)	71 (63; 76)	0.106
Hypertension	15 (75.0)	24 (80.0)	0.676
Smoker	1 (5.0)	4 (13.3)	0.336
Hyperlipoproteinemia	6 (30.0)	15 (50.0)	0.160
Diabetes mellitus	1 (5.0)	4 (13.3)	0.336
History of CVD	0 (0.0)	3 (10.0)	0.145
History of CHD	1 (5.0)	5 (16.7)	0.214
History of antiplatelets	4 (20.0)	9 (30.0)	0.430
History of anticoagulants	1 (5.0)	0 (0.0)	0.216
eGFR (ml/min per 1.73 m^2^)	86.9 (65.4; 98.2)	70.7 (61.7; 86.6)	0.104
NIHSS on admission	9 (6; 15)	n.a.	–
mRS 90 days	3 (2; 4)	n.a.	–
Deep location of ICH	15 (75.0)	n.a.	–
IVH extension	2 (10.0)	n.a.	–
Hematoma volume (ml)	10.3 (3.1; 24.4)	n.a.	–
Hematoma enlargement	5 (25.0)	n.a.	–
Perihematomal edema (ml)	23.0 (11.9; 61.5)	n.a.	–
Cytotoxic edema	9 (45.0)	n.a.	–

Data are presented as numbers (percentages) or median (interquartile range). *P* < 0.05 was considered statistically significant

*CHD* coronary heart disease, *CVD* cerebrovascular disease, *eGFR* estimated glomerular filtration rate, *IVH* intraventricular hematoma, *mRS* modified Rankin Scale, *NIHSS* National Institutes of Health Stroke Scale

### Temporal pattern of dimethylarginines after acute ICH

**Fig. 1:**
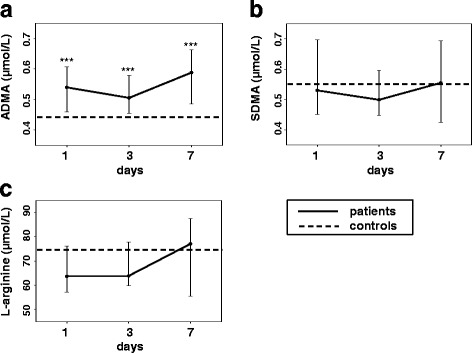
**a**–**c** Time courses of ADMA, SDMA, and L-arginine in acute ICH. Data are presented as median (interquartile range). Values in controls are presented by dashed line. Differences between patients and controls: *** *p* ≤ 0.001. Within group comparisons of marker levels between initial (≤ 24 h) and follow-up time points: significant differences were detected for ADMA (≤ 24 h versus 7 days; *p* = 0.030) and SDMA (≤ 24 h versus 3 days; *p* = 0.029)

In controls ADMA levels were 0.438 μmol/L (interquartile range 0.389–0.476), SDMA levels were 0.551 μmol/L (interquartile range 0.465–0.631), and L-arginine levels were 74.65 μmol/L (interquartile range 63.70–91.43). Levels of ADMA, SDMA, and L-arginine with interquartile range for ICH patients are shown in Fig. 1a–c. ADMA levels were significantly increased at any time point in ICH patients compared to controls (≤ 24 h, *p* < 0.001; 3 days *p* = 0.001; 7 days, *p* > 0.001), while SDMA and L-arginine levels were not (Fig. 1a–c). However, L-arginine showed a trend for lower levels at ≤ 24 h (*p* = 0.060) and 3 days (*p* = 0.063).

The binary logistic regression analysis with co-variables age, and eGFR showed a significant elevation of ADMA levels at days 1, 3, and 7 in ICH patients compared to levels in controls (ADMA ≤ 24 h, *p* = 0.003; ADMA 3 days, *p* = 0.005; ADMA 7 days *p* = 0.004).

ADMA levels increased after the initial time point (≤ 24 h) until day 7 (*p* = 0.030) (Fig. 1a). SDMA levels decreased between the initial time point (≤ 24 h) and day 3 (*p* = 0.029) (Fig. 1b). No significant differences were seen for L-arginine levels in regard to the temporal evolution (Fig. 1c).

### Dimethylarginines in relation to outcome

Patients were grouped according to mRS at 90 days as favorable (mRS 0–2) and unfavorable (mRS 3–6) outcome. Nine patients had favorable outcome, and 11 patients had unfavorable outcome. Outcome groups did not differ in regard to baseline characteristics and cardiovascular risk factors.

**Fig. 2:**
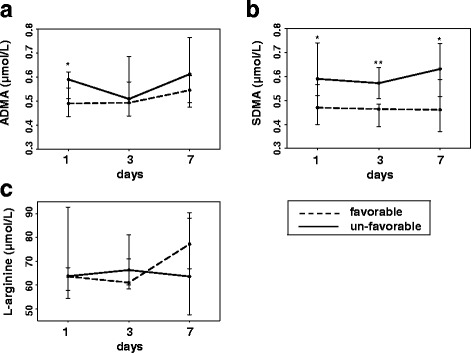
**a**–**c** Comparison of time courses of ADMA, SDMA, and L-arginine after acute ICH in patients with favorable and unfavorable outcome. The data are presented as median (interquartile range). Differences between outcome groups: *p ≤ 0.05; **p ≤ 0.01

ADMA levels ≤ 24 h were significantly higher in patients with unfavorable than in patients with favorable outcome (*p* = 0.031) (Fig. 2a). SDMA levels at any time point were significantly elevated in patients with unfavorable outcome compared to those with favorable outcome (SDMA ≤ 24 h, *p* = 0.016; SDMA at 3 days, *p* = 0.004; SDMA at 7 days, *p* = 0.031) (Fig. 2b). For L-arginine, no significant differences were detected in regard to outcome groups (Fig. 2c).

The binary logistic regression analysis did not confirm increased ADMA levels ≤ 24 h in patients with unfavorable outcome (*p* = 0.059). Increased SDMA levels ≤ 24 h (*p* = 0.048) and at 3 days (*p* = 0.028) but not at 7 days (*p* = 0.122) remained significantly associated with unfavorable outcome.

**Fig. 3:**
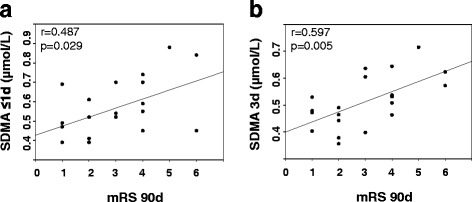
**a**, **b** Correlation of SDMA ≤ 1 and 3 days with mRS at 90 days

Correlation analysis revealed a significant correlation of SDMA levels at ≤ 24 h and 3 days with mRS at 90 days, whereas SDMA at 7 days and ADMA levels ≤ 24 h only tended to correlate with mRS at 90 days (ADMA ≤ 24 h, *p* = 0.088; SDMA ≤ 24 h, *p* = 0.029; SDMA at 3 days, *p* = 0.005; SDMA at 7 days, *p* = 0.081) (Fig. 3a, b). At other time points, no association between ADMA, SDMA, or L-arginine and mRS at 90 days was detected.

### Dimethylarginines in relation to secondary brain injury in ICH

Hematoma enlargement occurred in five patients and cytotoxic edema in nine patients (Table 1). To test the hypothesis that early levels of dimethylarginines (≤ 24 h) are associated with secondary brain injury, patients were grouped according to the median of hematoma and perihematomal edema volume as well as according to the occurrence of hematoma enlargement and cytotoxic edema. Of note, these outcome groups did not differ in regard to baseline characteristics and cardiovascular risk factors.

In patients with higher hematoma volume (> median; *n* = 10), SDMA levels but not ADMA levels (≤ 24 h) were significantly higher compared to those with smaller hematoma volume (< median; *n* = 10) (*p* = 0.029). ADMA levels (≤ 24 h) were significantly higher in patients developing hematoma enlargement (*n* = 5) compared to patients without hematoma enlargement (*n* = 13) (*p* = 0.003). SDMA levels (≤ 24 h) were significantly higher in patients with larger perihematomal edema volume (> median; *n* = 10) compared to those with smaller perihematomal edema volume (< median; *n* = 10) (*p* = 0.023), while perihematomal edema mostly consists of vasogenic edema, in part of the patients cytotoxic edema, could be detected. In patients with occurrence of cytotoxic edema (*n* = 9), ADMA and SDMA levels (≤ 24 h) did not differ from those in patients without cytotoxic edema (*n* = 11).

## Discussion

Our data show that (i) levels of ADMA are increased after acute ICH, (ii) higher levels of SDMA are associated with poor functional outcome, (iii) ADMA is associated with the occurrence of hematoma enlargement, and (iv) SDMA is associated with large hematoma and perihematomal edema volumes.

After ICH, inflammation, cellular damage, and proteolysis cause oxidative stress, which is known to increase protein arginine methyltransferase (PRMT) activity and decrease dimethylarginine dimethylaminohydrolase (DDAH) activity leading to elevated ADMA levels [20, 21]. In our study, ADMA levels but not SDMA levels were increased at each time point in ICH patients compared to controls. Of note, Wanby et al. reported no increase of ADMA levels in 22 ICH patients [12]. But the design of both studies seems barely comparable. Wanby and colleagues took only one blood sample at an undefined time point during the first days after admission. However, in our study, ADMA levels were increased at each of the specified time points during the first week after the event. Therefore, differences in study results might not be related to the blood sampling schedule but to the patient cohort. We demonstrated an increase of ADMA in patients with poor outcome in the univariate analysis, while after adjustment for co-variables ADMA levels only tended to be associated with unfavorable outcome. Since Wanby et al. did not report clinical severity or outcome for their patients, it cannot be discussed if the difference between the study results might be due to differences in ICH severity. An association of ADMA levels with outcome might be explained by the larger extent of damage inducing more oxidative stress. Importantly, this hypothesis is supported by elevated ADMA levels in patients with hematoma enlargement. However, it remains unclear if ADMA is directly involved in the pathophysiology of hematoma enlargement. ADMA may trigger oxidative stress via uncoupling of endothelial NOS (eNOS) and iNOS resulting in production of the toxic compounds superoxide (O_2_·^−^) and peroxynitrite (ONOO·^−^) [22, 23]. This may lead to further damage of the blood-brain barrier (BBB) facilitating hematoma enlargement. However, so far, direct evidence for ADMA-induced uncoupling of eNOS and iNOS is lacking for the cerebral circulation.

The continuous increase in ADMA levels between day 1 and day 7, as shown in our patient cohort, could possibly be explained by massive inflammation, proteolysis, and oxidative stress during the first days after ICH. Of note, follow-up data from a fraction of the patient cohort suggest that ADMA levels might be still elevated at day 90, although levels tended to decrease compared to those on day 7 (data not shown). However, so far, the length of and the reason for prolonged elevation of ADMA levels after ICH remain unclear.

Wanby et al. demonstrated lower levels of L-arginine in ICH patients than in controls [12]. In our study L-arginine––levels were also lower in the ICH patients than in the controls until day 3 after the event, but the level of significance was missed. Under conditions of L-arginine depletion, neuronal NOS (nNOS) synthesizes O_2_.^−^ [24]. Therefore, it is suggested that lower levels of L-arginine in ICH patients result in increased O_2_.^−^ production, potentially harming the zone of hypoperfusion surrounding the hematoma. Interestingly, addition of ADMA inhibited the nNOS-derived O_2_.^−^ production [24]. This might indicate a beneficial effect of ADMA after ICH, potentially limiting the pathology of BBB damage. Another beneficial effect of ADMA might be reduction of perfusion by increase of vascular tone to lower the volume of bleeding. In healthy subjects, infusion of ADMA with 0.10 mg/kg/min significantly decreased cerebral blood flow [25].

These ADMA effects might be pronounced in patients with poor outcome and hematoma enlargement since in these patients, ADMA production following cellular damage and proteolysis is particularly high. However, since these secondary complications are particularly detrimental and cause poor outcome, obviously any discussed beneficial ADMA effect might not be sufficient.

After ICH, elevated ADMA levels might also affect systemic blood pressure. Experimental data in rats showed that microinjection of ADMA in the rostral ventrolateral medulla reduced NO synthesis and increased blood pressure [26]. Importantly, high blood pressure after ICH has been associated with poor clinical outcome and secondary complications such as hematoma enlargement [27]. In addition, high blood pressure variability is associated with unfavorable outcome, possibly due to secondary injury of hypoperfused tissue affected by perihematomal edema [28]. However, optimized clinical management such as early intensive blood pressure-lowering is still discussed [29]. In particular, any treatment directly influencing NO levels cannot be recommended due to insufficient evidence. Recently, a systematic review has investigated safety and efficacy of the NO donor transdermal glyceryl trinitrate when applied after stroke. Patients showed blood pressure lowering, but outcome was not ameliorated [30]. Of note, the analyzed studies included both ischemic and hemorrhagic stroke patients.

SDMA levels were not different between ICH patients and controls in the current study and in the study of Wanby and colleagues [12]. Importantly, the current study showed a significant association of SDMA levels with outcome at 90 days. Giving a hint that SDMA is involved in the pathophysiology after acute ICH, SDMA levels measured in blood samples from the first day after the event were significantly higher in patients with larger compared to those with smaller hematoma and perihematomal edema volume. However, studies explaining these associations are so far missing in acute ICH patients and only data from other pathologies can be discussed. Recently, Feliers et al. demonstrated in glomerular endothelial cells, that SDMA causes eNOS uncoupling [31], a mechanism resulting in increased ROS production. It remains unclear, whether SDMA leads to eNOS uncoupling also in acute ICH. In another study, SDMA triggered ROS synthesis of monocytes by modulation of store-operated calcium channels [11]. Another mechanism, by which SDMA may add to poor outcome after ICH could be via pro-inflammatory effects.

Inflammation during the acute stage of ICH is of particular importance. Recently, leukocyte counts and the neutrophil-to-lymphocyte ratio on admission have been shown to be associated with functional outcome in ICH patients potentially contributing to neurological deterioration via edema formation [32]. Interestingly, also in ischemic stroke patients who developed parenchymal hematoma or in patients with symptomatic ICH after thrombolytic treatment, the neutrophil-to-lymphocyte ratio is increased [33]. SDMA might be involved in increased inflammation after ICH since in monocytes, it triggers nuclear factor kappa-light-chain-enhancer of activated B cell (NF-kappaB) activation and tumor necrosis factor-alpha (TNF-alpha) and interleukin-6 (IL-6) expression [34]. However, to our knowledge the association between dimethylarginines and inflammation in ICH patients has not been studied so far.

### Limitations

This study was the first to investigate the temporal pattern of dimethylarginine levels after ICH and their association with clinical outcomes. Due to the small number of patients included, it is necessary to confirm the results in a larger study. In addition, there is no evidence that the demonstrated association of dimethylarginines with functional and radiological outcome reflects a causative effect of these mediators in the pathophysiology of ICH. However, in the current study, elaborated serial MRI imaging provided detection of radiological outcome markers which cannot be achieved in routine clinical care. Thereby, the association of dimethylarginine levels with hematoma enlargement and perihematomal edema could be investigated. To confirm these data, a step from bedside to bench to elucidate the underlying mechanisms in experimental studies is warranted.

## Conclusions

ADMA and SDMA levels are increased after the acute event of ICH in relation to outcome and might be––though differentially––involved in secondary brain injury such as hematoma enlargement and perihematomal edema. However, further studies are needed to elucidate the mechanisms behind to investigate if there is any causal relationship between dimethylarginine levels, inflammatory response to, and outcome of intracerebral hemorrhage.

## Abbreviations

ADC: Apparent diffusion coefficient
ADMA: Asymmetric dimethylarginine
BBB: Blood-brain barrier
CHD: Coronary heart disease
CT: Computed tomography
CVD: Cerebrovascular disease
DDAH: Dimethylarginine dimethylaminohydrolase
DWI: Diffusion weighted imaging
eGFR: Estimated glomerular filtration rate
eNOS: Endothelial nitric oxide synthase
HE: Hematoma enlargement
HPLC–MS–MS: High-performance liquid chromatography–tandem mass spectrometry
ICH: Intracerebral hemorrhage
IL-6: Interleukin-6
iNOS: Inducible nitric oxide synthase
IVH: Intraventricular hematoma
MRI: Magnetic resonance imaging
mRS: Modified Rankin Scale
NF-kappaB: Nuclear factor kappa-light-chain-enhancer of activated B cells
NIHSS: National Institutes of Health Stroke Scale Score
nNOS: Neuronal nitric oxide synthase
NOS: Nitric oxide synthase inhibitor
ONOO^.−^: Peroxynitrite
O_2_.^−^: Superoxide
PRMT: Protein arginine methyltransferase
ROI: Region of interest
ROS: Reactive oxygen species
SAH: Subarachnoid hemorrhage
SDMA: Symmetric dimethylarginine
TNF-alpha: Tumor necrosis factor-alpha
